# Angiogenesis in Gynecological Cancers: Role of Neurotrophins

**DOI:** 10.3389/fonc.2019.00913

**Published:** 2019-09-19

**Authors:** Maritza P. Garrido, Ignacio Torres, Margarita Vega, Carmen Romero

**Affiliations:** ^1^Laboratory of Endocrinology and Reproductive Biology, Hospital Clínico Universidad de Chile, Santiago, Chile; ^2^Departamento de Obstetricia y Ginecología, Facultad de Medicina, Universidad de Chile, Santiago, Chile

**Keywords:** gynecological cancers, angiogenesis, VEGF, BDNF, NGF

## Abstract

Angiogenesis, or generation of new blood vessels from other pre-existing, is a key process to maintain the supply of nutrients and oxygen in tissues. Unfortunately, this process is exacerbated in pathologies such as retinopathies and cancers with high angiogenesis as ovarian cancer. Angiogenesis is regulated by multiple systems including growth factors and neurotrophins. One of the most studied angiogenic growth factors is the vascular endothelial growth factor (VEGF), which is overexpressed in several cancers. It has been recently described that neurotrophins could regulate angiogenesis through direct and indirect mechanisms. Neurotrophins are a family of proteins that include nerve growth factor (NGF), brain-derived growth factor (BDNF), and neurotrophins 3 and 4/5 (NT 3, NT 4/5). These molecules and their high affinity receptors (TRKs) regulate the development, maintenance, and plasticity of the nervous system. Furthermore, it was recently described that they display essential functions in non-neuronal tissues, such as reproductive organs among others. Studies have shown that several types of cancer overexpress neurotrophins such as NGF and BDNF, which might contribute to tumor progression and angiogenesis. Besides, in recent years the FDA has approved the use of pharmacologic inhibitors of pan-TRK receptors in patients with TRKs fusion-positive cancers. In this review, we discuss the mechanisms by which neurotrophins stimulate tumor progression and angiogenesis, with emphasis on gynecological cancers.

## Introduction: Angiogenesis in Gynecological Malignancies

Gynecological neoplasms belong to a group of malignances that include ovarian, cervical, uterine, fallopian tubes, vulvar, vaginal cancer and gestational trophoblastic neoplasms. The following sections of this review will be focused on the first two types, which are the most frequent (1). Gynecological neoplasms are characterized by exacerbated angiogenesis (which is defined as the generation of new blood vessels from pre-existing ones) and vascular endothelial growth factor (VEGF) is the most widely studied angiogenic factor in the context of cancer. VEGF is secreted by most tumor cells, mainly in response to hypoxia and low nutrient concentrations (2), and promotes angiogenesis through its receptors expressed in endothelial cells. This antecedent has been crucial for the development of new drugs as bevacizumab, a humanized monoclonal antibody directed against human VEGF. Unfortunately, this drug has shown modest results (3), because ovarian and uterine cells may overexpress other molecules that can act as angiogenic factors, such as neurotrophins (NTs) and their receptors (4–7).

NTs are a group of molecules widely present in the central and peripheral nervous system. They have a key role in developmental neurobiology, by regulating neuronal survival, differentiation, neurites growth, and synthesis of neurotransmitters (8). NTs not only display key roles in neuronal tissues, but also in several non-neuronal tissues, such as mammary glands (9, 10) and gynecological organs (11–13). During the neoplastic processes, NTs and their receptors are overexpressed by tumoral cells, promoting progression and angiogenesis in several cancer models. For instance, the expression of NTs predicts poor survival rates in breast and ovarian cancer patients (14–16) and NTs have been proposed as potential therapeutic targets in these neoplasms (4, 17, 18).

Angiogenesis is a key process to supply nutrients and oxygen to tumor cells, as well as a way for cells to leave or enter to the circulation (19). In fact, tumors that have a high microvascular density could be more aggressive and generate distant metastasis (20). The term angiogenesis was first used by the British surgeon John Hunter in 1787; however, the study of vascular morphology in animal and human tumors began only in the first half of twentieth century (21).

Endothelial cells, a baseline membrane and pericytes are the minimal components of vasculature. Endothelial cells form a barrier that controls the trans-endothelial flux of soluble components and most cell types (22). During angiogenesis, there are several important steps: a detection of humoral paracrine signals or angiogenic factors, resulting in the sprouting of endothelial cells, followed by an orchestrated increase of endothelial cell proliferation, migration, and differentiation (23). Activation of endothelial cells is accompanied by pericytes detachment, proliferation, and migration into the vessel interstitium to envelop the surface of the vascular tube. In addition, fibroblasts and endothelial cells build and remodel the new extracellular matrix (23, 24). All of these changes are necessary to generate new capillary vessels.

## Tumor Angiogenesis

Tumor growth has two phases: an avascular stage (when tumors are constrained at diameters of 1–2 mm) and a posterior vascular stage (25), in which tumor cells need to secrete soluble factors to promote an increase of angiogenesis and continued growing (26).

In the normal vasculature, endothelial cells are stable; rarely they sprout or divide and they are associated to mural cells (pericytes) in a basal membrane. However, in the case of the tumor vasculature several chromosomal abnormalities arise (27–29), as well as variations of size and thickness, irregular shape, and big trans-cellular holes and fenestrae (30, 31). These characteristics produce a decrease of blood flow and drug delivery, and increase the interstitial fluid pressure, the extravasation of blood components and the intravasation of tumor cells (30, 32). Particularly in gynecologic neoplasms, angiogenesis plays a key role, since the ovary and uterus cyclically regulate the angiogenesis during the ovarian cycle involving blood vessel growth and regression, with a fine regulation (33–35). Therefore, angiogenesis is undoubtedly crucial in gynecological cancers, but this process is uncontrolled. Given that angiogenesis is a complex process that involves different cell types, *in vivo* experiments constitute the ideal condition to evaluate it. Some examples of *in vivo* assays are: the chick embryo chorioallantoic membrane (CAM) assay (36), zebrafish embryo assay (37, 38), corneal micropocket assay (39, 40), and matrigel plug assays (41). Moreover, there are some experimental approaches *in vitro* to evaluate the angiogenic potential of cells, which may have some advantages, such as the reproducibility and low cost to perform these assays (42). However, it is considered that *in vitro* assays evaluate vasculogenesis or *de novo* formation of vasculature-like structures and usually involve only endothelial cells and extracellular matrix. Examples of this are tubular formation assays in matrigel (43, 44) and the recently developed microfluidic cell culture systems (45). Nevertheless, *in vitro* assays are widely used, because they are a cheap and reproducible method to evaluate the angiogenic potential (46).

## VEGF: Classical Angiogenic Factor in Cancer

There are many known angiogenic factors, among which VEGF is the most widely studied in the context of cancer. VEGF genes include VEGF-A to VEGF-E and another related gen, placental growth factor (PLGF) (47–50). VEGF-A (from now referred as VEGF) has the most important effect in the formation of blood vessels during development or in pathological conditions as cancer (51). At the same time, VEGF undergoes alternative exon splicing (52, 53), leading to several transcripts that include VEGF_121_, VEGF_145_, VEGF_165_, VEGF_189_, and VEGF_206_, which give origin to VEGF peptides of 121, 145, 165, 189 and 206 amino acids, respectively (54). Besides, VEGF_121_ is totally secreted and VEGF_165_ is partially secreted from cells (55, 56). In ovarian, endometrial and cervical cancers, VEGF_121_ and VEGF_165_ are the most dominantly expressed (57–60).

## Role of Neurotrophins in Gynecological Cancer Angiogenesis: NGF/TRKA and BDNF/TRKB

### Neurotrophins and Its Functions in Reproductive Tissues

**Figure 1:**
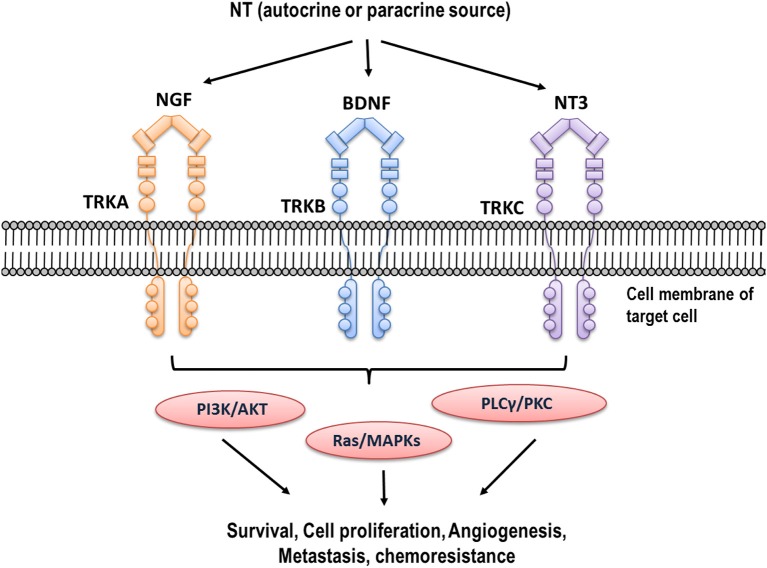
Neurotrophins and their high affinity receptors. Different NTs (NGF, BDNF, NT3) are expressed in high concentration in tumor cells. They bind to their high affinity receptors (TRKA, TRKB, and TRKC, respectively), producing trans-phosphorylation of tyrosine residues of intracellular domain and activating different signaling pathways such as PI3K/AKT, MAPK/ERK, and PLCγ/PKC.

NTs belong to a family of homodimeric polypeptide growth factors that promote neuronal survival and differentiation, and display important functions in non-neuronal cells (13, 61). Members of the NTs family include nerve growth factor (NGF) that was first described by Dr. Levi-Montalcini in 1956 (62), brain derived neurotrophic factor BDNF, neurotrophin-3 (NT-3), and neurotrophin-4/5 (NT-4/5) (63). Among them, NGF and BDNF are the most important NTs studied in the context of reproduction and cancer. NTs bind with different affinity to Tropomyosin kinase (TRK) receptors and produce the dimerization and transphosphorylation of its tyrosine kinase domains, activating PI3K/AKT, MAPK/ERK, and PLCγ/PKC signaling pathways (64). NGF binds with high affinity to TRKA receptor, while BDNF binds preferentially to TRKB receptor (PMID: 1649702, PMID: 2927393), as shown in Figure 1.

Both NGF/TRKA and BDNF/TRKB are expressed in reproductive tissues as the ovary and uterus (13, 65). These NTs are involved in the control of early follicular growth and ovarian function (66–70). NGF increases cell proliferation of granulosa and thecal cells and promotes the expression of Follicle Stimulating Hormone (FSH) receptor in rat and human granulosa cells (68, 71, 72), while BDNF/TRKB are required for the growth of newly formed follicles and are involved in the maturation of human oocytes and their developmental competence after fertilization (70, 73, 74). In addition, BDNF levels in follicular fluid (75) and plasma (76) have been studied as possible predictors of *in vitro* fertilization outcome. BDNF and NGF seem to have a positive correlation with oocyte maturation and pre-implantation and with embryonic development in various mammalian species, including humans (73, 77–80).

On the other hand, NGF expression is present in epithelial and stromal cells in the rabbit uterus (81), as well as in human uterus (82), but its expression is lower than in the ovary (13). In addition, NGF expression seems to be necessary to ensure maternal tolerance in healthy pregnancies in mice, but an excess of NGF results in fetal rejection due to exacerbated inflammation (83). BDNF levels in menstrual blood are higher than in peripheral blood, and this factor is also present in the endometrium in both follicular and luteal phases (65). Furthermore, BDNF levels in menstrual blood of fertile women are higher than in anovulatory women (65). All these findings show that NGF and BDNF play a key role in the homeostasis and function of tissues in the context of female reproduction.

### Roles of Neurotrophins as Direct and Indirect Angiogenic Factors

**Figure 2:**
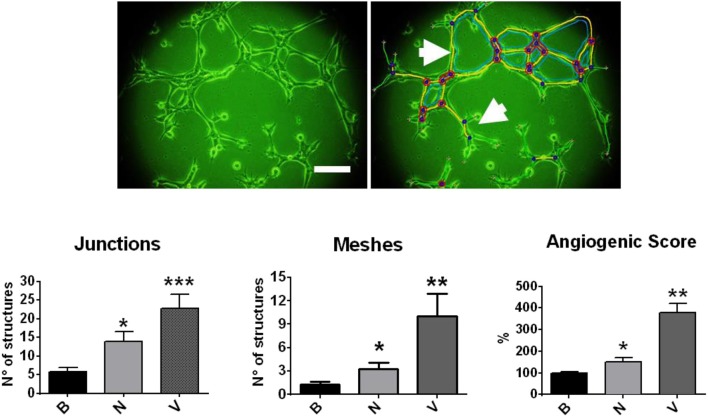
Effect of NGF and VEGF in a tubular formation assay in matrigel with EA.hy926 cells. Cells were disposed in matrigel and stimulated with NGF (N) and VEGF (V). Upper picture: photography of EA.hy926 cells (4 h of assay), which was analyzed by Image J Angiogenesis Analyzer. Bar charts were obtained from multicellular junctions and polygonal structures or meshes, as the arrows indicate. ^*^p < 0.05; ^**^p < 0.01; ^***^p < 0.001, according to Kruskal Wallis test. Figure obtained from Supplementary Material of Garrido et al. (87) (permission has been obtained).

One of the first evidence of the angiogenic role of NGF comes from the expression of TRKA receptors in human umbilical vein endothelial cells (HUVEC): when using a VEGF-neutralizing antibody, NGF-induced HUVEC proliferation was not observed (84). In another work, NGF from different biological sources (mouse, viper and cobra) was tested in a CAM assay (85), and an increased rate of angiogenesis in a dose-dependent fashion and comparable with recombinant VEGF effects was described. Additionally, one study performed in matrigel plugs in immune-deficient mice shows that NGF strongly increases invasion, cord formation and the monolayer permeability of endothelial cells (86). Furthermore, a recent work shows that NGF increases cell proliferation, migration and differentiation of the human endothelial cell line EA.hy926 in a dose-dependent manner (87). In fact, Figure 2 shows that NGF increases inter-cellular contact structures (junctions) and polygonal structures (meshes) of EA.hy926 cells, evaluated by Image J Angiogenesis Analyzer (88). Additionally, it has been reported that NGF increases the angiogenic score of EA.hy926 cells, the effect being several times lower compared with VEGF (87).

In a comparable way, BDNF displays direct angiogenic effects in other types of tissues. For example, in a model of BDNF null mice, the survival of endothelial cells in intra-myocardial arteries and capillaries in the early postnatal period is impaired (89). Additionally, BDNF increases angiogenic tube formation of the endothelial cells in HUVEC (90). Besides, the overexpression of BDNF in a mouse endothelial cell line promotes endothelial cell proliferation, migration, invasion and survival (91). This evidence indicates that BDNF/TRKB exhibits a direct role in the angiogenic process and can partially explain that the anti-angiogenic therapy with Bevacizumab (neutralizing antibody against VEGF) is not optimal in the cancer context.

On the other hand, both NTs (NGF and BDNF) have an indirect angiogenic role, mediated by VEGF modulation in different cellular models. It is described that NGF and BDNF induce VEGF expression in MAPK/ERK 2-dependent pathways in granulosa cells (92) and osteoblasts (93), respectively. Besides, NGF promotes VEGF expression in neuronal superior cervical ganglia (94), while BDNF increases VEGF expression in human chondrosarcoma (95) and neuroblastoma cells (96). Another key point is that plasmatic levels of VEGF are lower in deficient BDNF animals compared to wild type animals (97). All these antecedents indicate that NTs not only act directly in vascular cells, but also affect several cell types by increasing VEGF expression and therefore their angiogenesis potential.

### Role of NGF/TRKA in the Ovarian Cancer Angiogenesis

Ovarian Cancer is the most lethal gynecological malignancy in developed countries (98–100). It is characterized by non-specific symptoms and therefore is diagnosed at later stages, resulting in poor survival rates (101, 102). Approximately 80% of them are Epithelial Ovarian Cancer (EOC) (101) which is characterized by its high extent of angiogenesis that facilitates rapid tumor growth and dissemination (103). NGF and its high affinity receptor TRKA are found in very low levels or are absent in normal ovarian surface epithelium, whereas they are highly expressed in EOC (60). Another study shows that significantly higher levels of NGF, total TRKA, and phospho-TRKA (active receptor) are present in poorly differentiated EOC vs. normal ovary (4). In addition, NGF/TRKA stimulates cellular proliferation of EOC cells, by the activation of MAPK/ERK and AKT pathways, increasing Bcl2/Bax ratio and c-Myc (104), indicating the importance of NGF/ TRKA in EOC progression and suggesting that they could be considered as a potential tumor markers. As previously shown, several studies performed in *in vitro* and *ex vivo* models support the direct angiogenic role of NGF in EOC (105). It is relevant to point out that the TRKA receptor is present in endothelial cells from EOC biopsies (4), supporting the idea that the endothelium can respond to NGF stimulation.

On the other hand, an indirect angiogenic role of NGF has been described through the modulation of VEGF expression in EOC. In fact, in EOC explants, NGF increases in a dose-dependent manner the mRNA of VEGF_121_, VEGF_165_, and VEGF_189_ (60). Equivalent results were obtained in *in vitro* models, where NGF increases VEGF expression and protein levels in the culture supernatants of the EOC cell line (4).

### Role of BDNF/TRKB in the Ovarian Cancer Angiogenesis

It has been reported that TRKB displays a key role in ovarian development, which gives proliferative signaling in granulosa cells during the beginning of mammalian ovary development (70). Increased TRKB levels can promote the increase of cell proliferation, invasion and angiogenesis, suppression of anoikis and decreased chemotherapy response and apoptosis in different cancer cell lines, including ovarian cancer cells (5, 106–112). Observational studies show that high TRKB expression in ovarian cancer is correlated with poor survival in ovarian cancer patients (5), and that TRKB is overexpressed in metastatic lesions compared with the corresponding primary lesions (113). In addition, BDNF treatment enhances cell invasion and migration of ovarian cell lines and TRKB-silenced cells increase the percentage of apoptotic cells (5). This evidence indicates that BDNF/TRKB may contribute to ovarian cancer progression.

In agreement with other authors, our group has found that TRKB receptor is present in stroma and in transformed epithelia of human ovary. The active TRKB receptor is upregulated in serous adenocarcinomas and its immunodetection is almost absent in the epithelia from functional ovaries or ovarian serous adenomas (114)

Interestingly, in ovarian cell lines, the silencing of TRKB receptor reduces VEGFR-2 mRNA by 70% (5), which suggests that BDNF could regulate the expression of VEGF receptors. In addition, a positive correlation between TRKB expression and lymph vessel density has been described in ovarian cancer (113). These results are consistent with other studies, in which BDNF promotes VEGF-C-dependent lymphangiogenesis in chondrosarcoma cells (95) and TRKB expression is associated with the expression of VEGF-C and VEGF-D in oral squamous cell carcinoma (115). These findings suggest that BDNF could be implicated in ovarian cancer progression and modulate angiogenesis and/or lymphangiogenesis by the increase of different VEGF isoforms.

## Role of NTs in Cervical Cancer and Uterine Pathologies

Cervical cancer is the fourth most frequent cancer in women. Approximately 90% of deaths from cervical cancer occur in low-income and middle-income countries, in which strategies of prevention, early diagnosis, effective screening, and treatment programs are less common (116).

In the context of cervical cancer, BDNF/TRKB are perhaps the best studied NTs. It has been described that BDNF and TRKB expression are significantly higher in cervical cancer tissues than in normal tissues and that their presence is higher in advanced stages of this neoplasm (6, 7). In addition, BDNF levels are positively associated with lymph node metastasis (7) in cervical cancer patients. In cervical cancer cell lines, BDNF/TRKB increases cell proliferation (7, 117), apparently involving ERK and AKT signaling pathways (118). TRKB downregulation in cervical cancer cells suppress the activation of epithelial mesenchymal transition (EMT) by downregulation of N-cadherin and vimentin, among other proteins, and strongly diminishes cell proliferation, migration and invasion (117, 118).

Considering that the activation of ERK signaling pathway by BDNF/TRKB was associated with an increase of VEGF expression in osteoblasts (93), and given that TRKB can activate PI3K and ERK signaling pathways which regulate VEGF expression in several models (119–121), it is plausible that the VEGF expression could be increased by TRKB in cervical cancer, similarly to ovarian cancer.

There is no direct evidence that overexpression of NTs and its receptors are involved in the physiopathology of endometrial cancer. However, antecedents suggest that NTs could contribute to this pathology, since their expression increase in endometriosis (122–124), a condition that has been associated with higher risk of ovarian and endometrial cancer (125–127). The endometriosis is an estrogen-dependent inflammatory disease, characterized by the presence of endometrial-like tissue outside the uterine cavity (128). An important characteristic of this pathology is that angiogenesis is deregulated. In endometriosis, the VEGF expression is increased and promotes the spreading of new blood vessels at the endometriotic lesions and surroundings, which contributes to the survival of lesions (129). A recent study has shown that drospirenone, a drug used for endometriosis treatment, significantly decreases inflammatory cytokines and NGF expression, as well as VEGF expression in human endometriotic stromal cells (130). Similarly, Ginsenoside (a ginseng-derivate extract) decreases both VEGF and BDNF in rat endometriotic implants (131). These antecedents suggest that NTs could contribute not only to the pelvic chronic pain typical of endometriosis, but also to pathological angiogenesis, probably by the increase of VEGF levels.

## Pharmacologic Inhibitors of Neurotrophin Receptors

Since the TRK receptors (TRKA, TRKB, and TRKC) are implicated in the progression of different kind of neoplasms, several drugs have been developed to target tumors that overexpress TRK receptors or present chromosomal rearrangements of TRK genes. For instance, in 2018, the Food and Drug Administration (FDA) approved Larotrectinib (Vitrakvi) for treatment of adult and pediatric patients with solid tumors that have TRK gene fusions (132). This was based in promissory results of 3 clinical trials (NCT02122913, NCT02637687, and NCT02576431) with Larotrectinib that showed an objective response rate of 75% in pediatric patients, with good tolerability and safety (133, 134). Larotrectinib is a small molecule that binds to NTs receptors, thereby preventing neurotrophin-TRK interaction and TRK activation, which results in the induction of cellular apoptosis and the inhibition of cell growth (135). It is important to point out that Larotrectinib was one of the first “tissue-agnostic drug” approved by FDA, concept that refers to a substance to treat cancer based on genetic and molecular features of tumor cells, regardless of the cancer type or origin (136).

Additionally, Entrectinib (Rozlytrek), a potent and selective ATP-competitive inhibitor, was approved by the FDA in 2019 for adults and pediatric patients above 12 years old with solid tumors (as ovarian cancer) that have a TRK fusion without a known acquired resistance mutation (137). The first results of phase I/II studies show promising results: for example, an objective response rate of 57.4% was obtained in 54 adults with advanced or metastatic TRK fusion-positive solid tumors (138). Unfortunately, some patients have reported resistance to TRK inhibition with this drug considered as first generation of TRK inhibitors (139), probably due to mutations in TRK domain (140, 141). To improve this aspect, a next-generation TRK-targeted agent is under study. For example, Loxo-195 is a recently developed drug, which phase 1/2 of the study started in 2017 in patients with TRK-positive solid tumors and TRK fusion-positive cancers (clinical trials NCT03215511 and NCT03206931). This drug could become an alternative treatment for tumors with acquired resistance to first-generation TRK-targeted agents (142). VMD-928 is another specific TRK inhibitor which is under phase 1 of the study since 2018 for treatment of advanced adult solid tumors or lymphoma (NCT03556228).

Because TRK overexpression is present in gynecological cancers, and particularly TRK fusion has been described in cervical and uterine cancer (143, 144), the use of TRK inhibitors could be beneficial in these kinds of neoplasms. However, it is necessary to continue the studies to determine their effectiveness in gynecological cancers.

## Conclusions

NGF/TRKA and BDNF/TRKB are the main NTs studied in the context of cancer. These NTs and their receptors are over-expressed in gynecological neoplasms, such as ovarian and cervical cancers, in which they promote the progression of these diseases. Furthermore, NTs are involved in uterine pathologies such as endometriosis, which suggests that they could contribute to endometrial cancer progression, however this has not been elucidated yet. NTs are indirect angiogenic factors, acting through the induction of VEGF expression in ovarian cancer cells; besides, it is possible that NTs could display the same effect in other cancer cells such as cervical and endometrial. In addition, NTs exhibit a direct angiogenic role, mainly studied in endothelial cells that express NTs receptors, and respond by increasing endothelial cell proliferation, migration and differentiation. Moreover, NTs increases angiogenesis both in *in vitro* and *in vivo* models. Consequently, NTs and their receptors may be considered as important angiogenic factors, mostly in the context of anti-angiogenic therapy against VEGF, where overexpression of NTs could increase the angiogenesis independent of VEGF levels and contribute to therapy failure. Since NTs and TRK receptors are drivers of a wide variety of adult and pediatric cancers as gynecological neoplasms, the FDA has recently approved pan-TRK inhibitors for the treatment of TRK fusion-positive solid tumors. Because TRK fusion has been described in several gynecological cancers, the recently developed TRK inhibitors emerge as a new therapeutic approach for the treatment in this subtype of neoplasms. Given that angiogenesis is a key feature in gynecological neoplasms, and NTs acts as direct and indirect angiogenic factors, it may be relevant to study whether TRK inhibitors could improve the efficacy of anti-angiogenic drugs as bevacizumab, which was not elucidated yet.

## Author Contributions

MG, IT, and CR: conceptualization. MG: writing original draft. MV and CR: writing, review, and editing.

### Conflict of Interest Statement

The authors declare that the research was conducted in the absence of any commercial or financial relationships that could be construed as a potential conflict of interest.
